# Hebra’s Continuous Tepid-Water Bath

**Published:** 1884-08

**Authors:** 


HEBRA'S CONTINUOUS TEPID-WATER BATH.

The continuous tepid-water bath occupies a prominent place among the elder Hebra's valuable contributions to general therapeutics. This bath consists of a large bath-tub, in which an iron frame with transverse slats is suspended by chains, so that the patient may be withdrawn from and returned to the water by means of a crank. The iron frame is covered with a horse-hair mattress, and furnished with comfortable pillows. The patient, perfectly nude—without any surgical dressing whatever—is placed in an easy position upon the mattress, and the bath-tub is filled with water of 30-31° C. temperature. A sensation of chilliness is usually experienced at this period, which is immediately dispelled by the rapid elevation of the water's temperature to 38-40° C. The temperature of the bath is permanently maintained at this degree by the continuous renewal of the water. A sheet, or other thin covering, may be thrown over the patient, and is useful by reason of its subjective effect.

The patient remains in the bath, day and night, except when withdrawn for the purpose of evacuation of the rectum or the bladder. There is no limit to the period of time which may be spent in the bath without detriment to the general health. Hebra has kept patients in the bath for periods of nine and ten months.

From this brief sketch it will be inferred that the pathological conditions which indicate the continuous tepid-water bath, are numerous and varied.

Burns, gangrenous wounds, bed-sores, and severe cases of pemphigus are notable examples of such conditions.

The best local treatment for burns,—especially when a considerable extent of surface is involved,—is to be found in the continuous bath. The terrible pain is relieved as by magic, psychic disturbance is quieted, and the patient frequently falls into a refreshing sleep. The intense *thirst*,—usually developed during the second stage,—is slaked, without the imbibition of liquids, which are rejected as soon as swallowed. Of course, in severe burns this method of treatment is too frequently of a merely palliative character; the patient dies in the bath just as he does under other methods of treatment.

When, however, death does not immediately follow the burn, we have a method of local treatment, at once simple, painless, and effective. "The water protects the wound surfaces from contact with the atmospheric air, prevents the decomposition of the sphacelus, and favors its separation; it makes every dressing and change of dressing unnecessary, limits the secretion of pus, and hastens cicitrization."—*Billroth.*

Gangrenous wounds,—especially the "puerperal ulcers" of the vulva and vagina, during the *puerperium*; bed-sores, from all causes, but particularly from spinal injuries, are influenced in the most favorable way. The patient is spared the distress and fatigue of all change of dressing, the wounds receive absolute antiseptic attention, and the attendants are relieved of much tedious and unnecessary labor.

Dermatologists very generally admit that grave cases of pemphigus find the best local treatment in Hebra's continuous bath. The pain, itching and burning sensations of this distressing affection are relieved, and comparative ease and comfort gained. In these cases the action of simple water is both palliative and curative.

As at present informed, there is no hospital in America in which the continuous tepid-water bath of Hebra is an accepted institution. It is difficult to give a good reason for such willful neglect of an invaluable therapeutic resource. The cost of construction and maintenance is relatively small, so that expense can not be pleaded as an extenuating circumstance.

A hospital, with a maximum capacity of ninety beds, has been recently constructed in Chicago. It is well endowed. Ten thousand dollars, we are told, has been recently expended upon surgical apparatus, and yet a case of universal burn cannot receive proper attention.

